# A case of IgA pemphigus, with a poor response to dapsone, successfully treated with adalimumab

**DOI:** 10.1002/ccr3.8807

**Published:** 2024-05-14

**Authors:** Saman Al‐Zahawi, Kambiz Kamyab, Kamran Balighi

**Affiliations:** ^1^ Department of Dermatology, Razi Hospital Tehran University of Medical Sciences (TUMS) Tehran Iran; ^2^ Department of Dermatopathology, Razi Hospital Tehran University of Medical Sciences (TUMS) Tehran Iran; ^3^ Autoimmune Bullous Diseases Research Center, Razi Hospital Tehran University of Medical Sciences Tehran Iran

**Keywords:** adalimumab, dapsone, IgA pemphigus, IgG‐driven pemphigus, subcorneal pustular dermatosis

## Abstract

**Key Clinical Message:**

IgA pemphigus is usually treated by Dapsone. Recalcitrant cases may be treated by Colchicine, Sulfapyridine, or Acitretin. Some patients with recurrent severe disease may not respond to the aforementioned medications. Our study highlights the role of TNFa inhibitor as an alternative modality in the treatment of recalcitrant IgA pemphigus.

**Abstract:**

IgA pemphigus is a rare autoimmune blistering disease characterized by a pruritic, annular, vesiculopustular eruption. In IgA pemphigus, there are IgA autoantibodies targeting the keratinocyte cell surface adhesion molecules, causing cell‐to‐cell dehiscence and a flaccid vesiculopustular eruption, mainly in the axilla and groin. Dapsone, despite being the drug of choice for treating IgA pemphigus, is not effective in clearing lesions in a minority of patients and such rare cases of recalcitrant IgA pemphigus need alternative modalities of treatment. Here, we report the successful treatment of a 50‐year‐old male patient with an adalimumab injection who had a poor response to dapsone.

## Graphical abstract

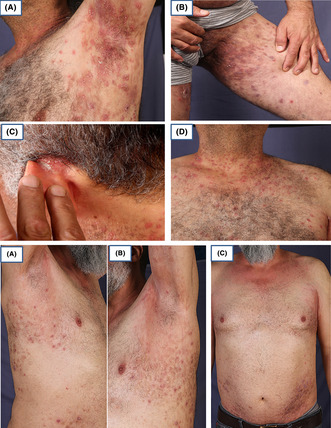

Fig 1: Multiple pustules on an erythematous base with areas of hyperpigmentation in Axilla (A), Groin (B), Left postauricular (C), and the neck.Fig 2: A remarkable response to Adalimumab 40mg/ every two weeks after four months from therapy. Left and right axilla (A) & (B), trunk (C)

## 1 INTRODUCTION

IgA pemphigus is a rare autoimmune blistering disease characterized by a pruritic, annular, vesiculopustular eruption. In IgA pemphigus, there are IgA autoantibodies targeting the keratinocyte cell surface adhesion molecules, causing cell‐to‐cell dehiscence and a flaccid vesiculopustular eruption.^1^

It occurs mainly in middle and elderly individuals. Clinically, it presents with crusted plaques in the axilla or groin, but the entire trunk and even the scalp may be involved.^2^ Histologically, there is neutrophilic infiltration of the epidermis leading to two distinct types of IgA pemphigus: intraepidermal neutrophilic type and subcorneal pustular type (SCP). Acantholysis of keratinocytes is not a feature of IgA pemphigus, unlike pemphigus vulgaris.^3^ Direct immunofluorescence (DIF) is required to establish the diagnosis of IgA pemphigus.

Although the course is milder than IgG‐driven pemphigus, dapsone is often administered to control lesions and prevent recurrence. It is wise to evaluate the patient from both hematological and infectious aspects, as there are reported cases of IgA pemphigus associated with IgA gammopathy, human immunodeficiency virus, and connective tissue diseases.^4^, ^5^ The aim of this manuscript is to report the successful treatment of IgA pemphigus with Adalimumab injection who had poor response to dapsone. A remarkable clearance of the lesions was observed after 4 months of Adalimumab injection/40 mg every 2 weeks. Long‐term follow‐up is needed to determine the time of tapering, the time of discontinuing the medication, and the recurrence rate.

## 2 CASE REPORT

**FIGURE 1:**
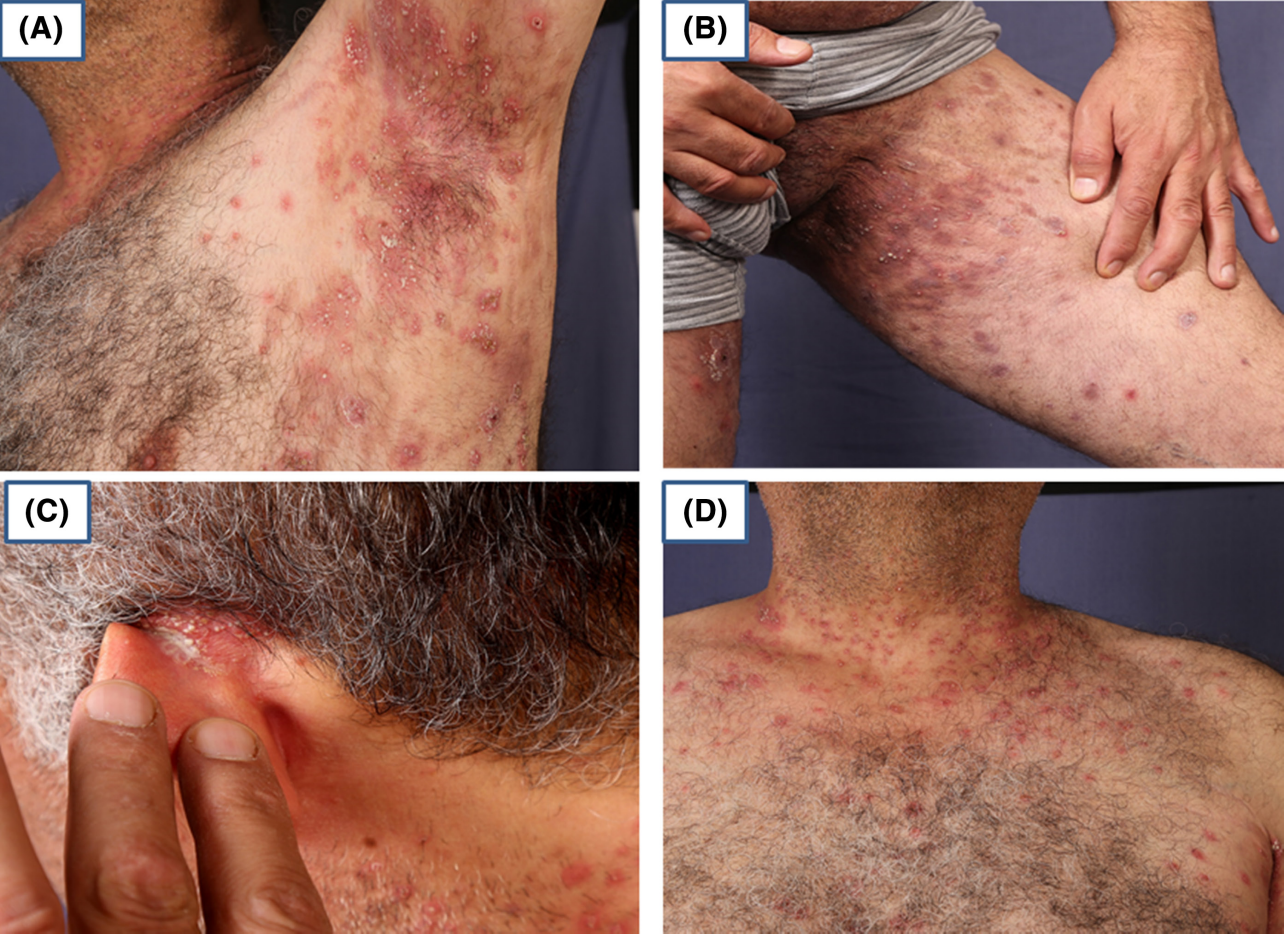
Multiple pustules on an erythematous base with areas of hyperpigmentation in Axilla (A), Groin (B), Left postauricular (C), and the neck (D).

**FIGURE 2:**
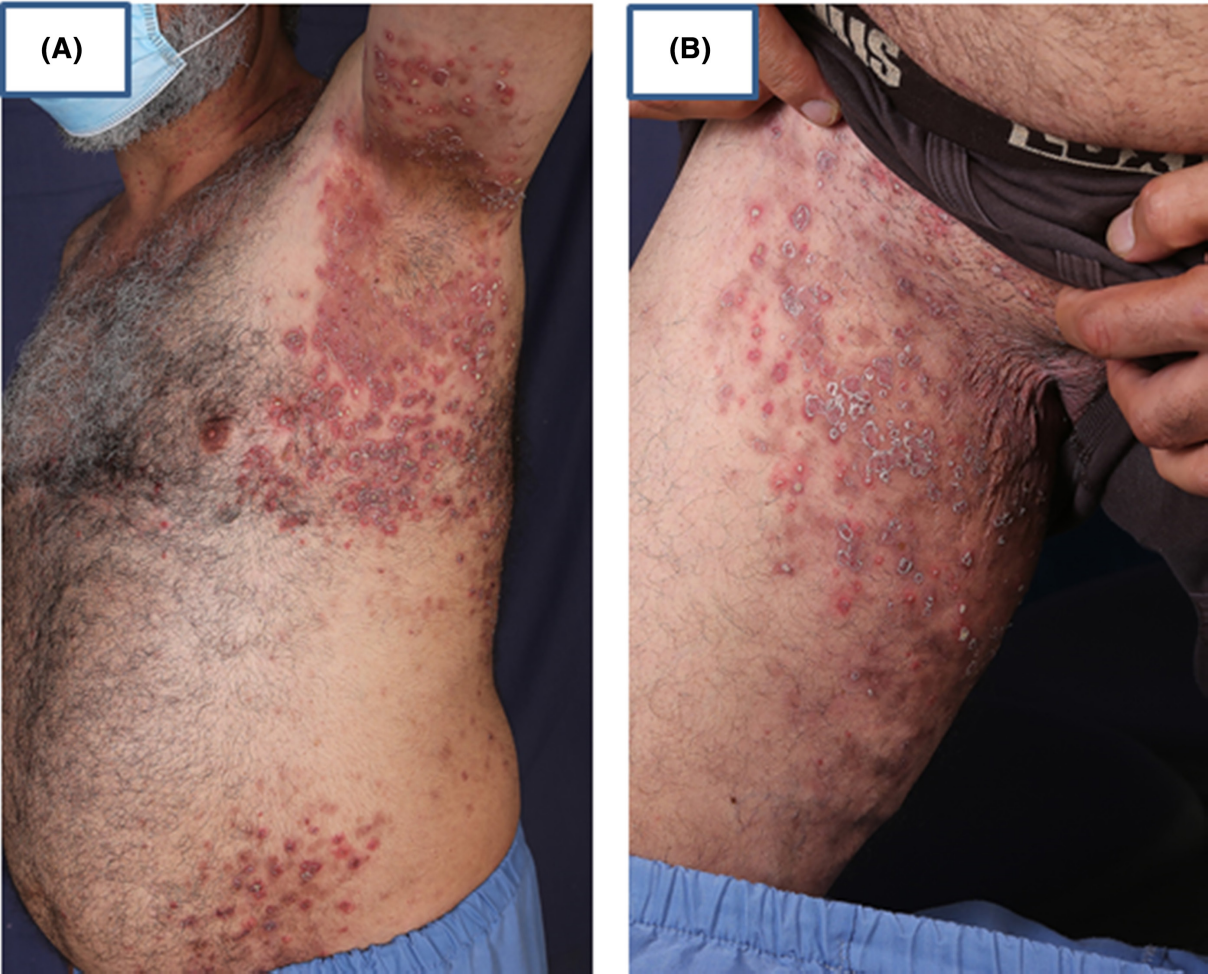
Persistent of the eruption and complete control of the lesions by dapsone therapy for 2 years, Axilla (A), Groin (B).

**FIGURE 3:**
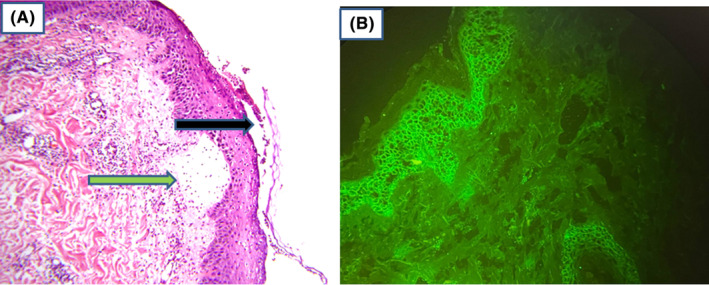
Specimen from abdomen showing subcorneal pustule that contains neutrophils (black arrow), there is also epidermal acanthosis with neutrophilic spongiosis. Edema of the papillary dermis with mixed lymphocytic, eosinophilic, and neutrophilic infiltration of the upper dermis (green arrow) could be observed (A). Direct immunofluorescence testing for IgA on perilesional skin showing intercellular deposits of IgA (B).

A 50‐year‐old male, with a known case of IgA pemphigus for nearly 10 years, with diffuse flexural involvement (Figure 1A–D), was under regular treatment with dapsone for 2 years with poor response (Figure 2A,B). Repeated histology and direct immunofluorescence (DIF) confirmed the diagnosis of the subcorneal pustular (SCP) type of IgA pemphigus (Figure 3A,B). The long‐period treatment with dapsone was unsuccessful in completely controlling the diffuse blisters and erosions in the flexural surfaces and trunk.

## 3 TREATMENT

**FIGURE 4:**
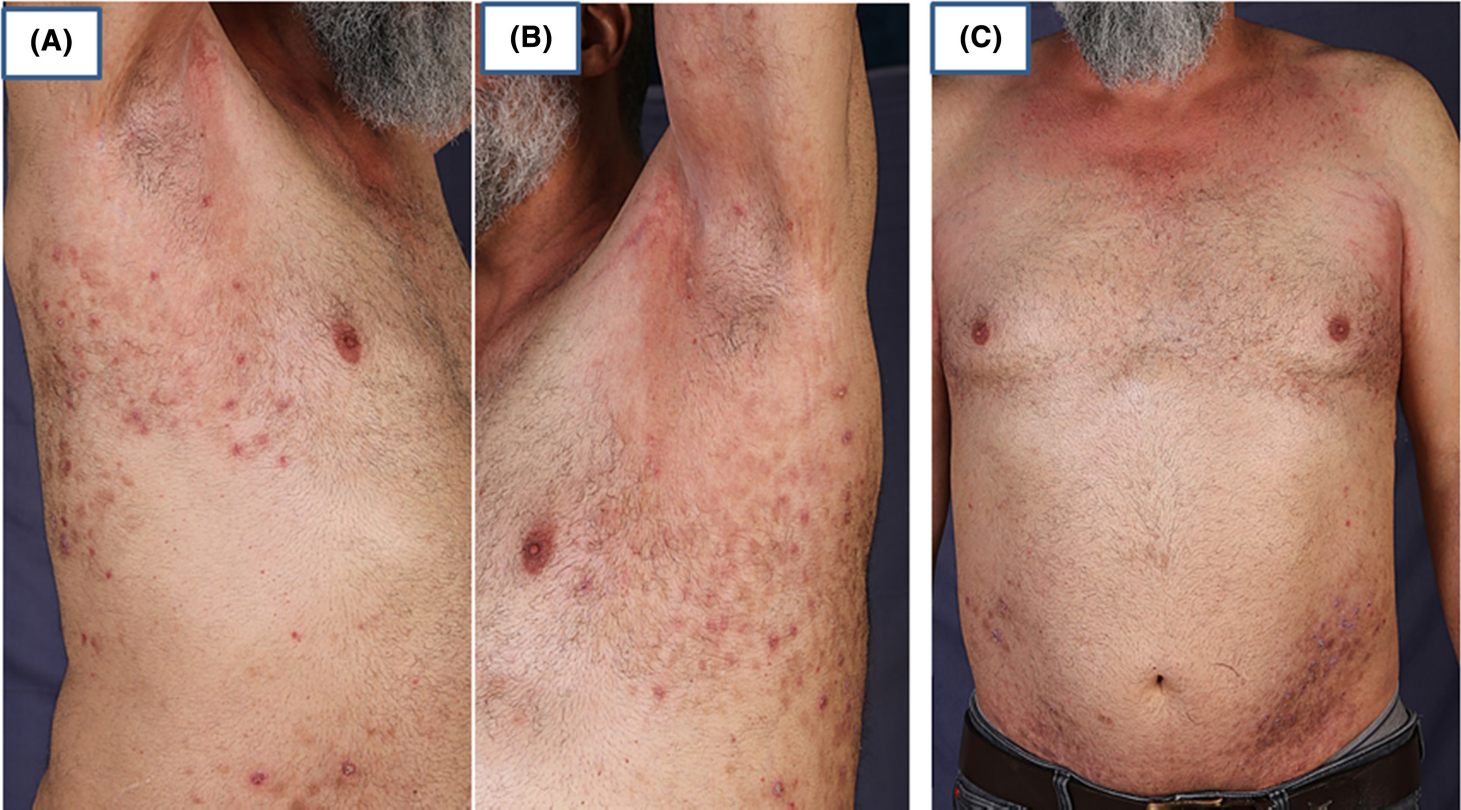
Remarkable response to adalimumab 40 mg/every 2 weeks after 4 months from therapy. Left and right axilla (A) and (B), trunk (C).

Adalimumab injection was initiated based on previous reports indicating the efficacy of TNF‐alpha inhibitors in controlling IgA pemphigus.^6^ Adalimumab was administered every 2 weeks for the last 2 years. Remarkable clearance was achieved after 4 months after starting the medication (Figure 4A–C). Tapering the medication or ceasing it leads to a rapid eruption of new lesions at the sites of previous old lesions with re‐adjustment of the dose to the usual 40 mg every 2 weeks for lesions clearance.

Because of the rarity of cases of recalcitrant IgA pemphigus treated with adalimumab and no clear guidelines to taper or discontinue the drug in the previously reported cases, the author recommends continuing adalimumab injection as in the case of dapsone treatment in IgA pemphigus until complete remission for a long time and then trying the chance of reducing the dose of the drug, with caution.

## 4 DISCUSSION

IgA pemphigus is a rare autoimmune blistering disease characterized by pruritic, annular, and vesiculopustular eruptions. In IgA pemphigus, there are IgA autoantibodies targeting the keratinocyte cell surface adhesion molecules causing cell‐to‐cell dehiscence and flaccid vesiculopustular eruption. Middle age and elderly persons are more commonly affected by IgA pemphigus but patients as young as 1 month have been reported.^7^

Although the etiology of IgA pemphigus is unknown, multiple associations have been reported. Perhaps the most important association is the association with IgA monoclonal gammopathy reported initially by Wallach et al., he observed this finding in 6 patients of a total of 29 patients with IgA pemphigus.^4^ Hiroshi et al also reiterated the coincidence of IgA pemphigus and multiple myeloma and raised concern about the association of IgA pemphigus with IgA‐type multiple myeloma.^5^ The introduction of COVID‐19 vaccination in the last 3 years was accompanied by different dermatological side effects; one of the reported dermatoses after COVID‐19 vaccination was IgA pemphigus.^8^ IgA pemphigus also has been reported in patients with ulcerative colitis and it seems that patients with ulcerative colitis respond less to the usual conventional treatment.^9^, ^10^ Three cases of IgA pemphigus have been reported in patients with HIV in which they responded very well to topical clobetasol and dapsone.^11^ Other reported associations with IgA Pemphigus include Rheumatoid arthritis and Sjogren syndrome.^1^

While there is no direct relationship between IgA pemphigus and the aforementioned diseases, it is wise to do complete hematological and infectious laboratory tests, which were normal in our case. Our patient had pruritic, localized, annular lesions in the groin, trunk, neck, and axilla which are the common usual sites of IgA pemphigus. These annular lesions are often painful and in rare cases may involve the whole trunk or have a similar clinical presentation to pemphigus vulgaris, in which sparing of the mucosa is in favor of IgA pemphigus.^12^

The clinical differential diagnosis for IgA pemphigus includes tinea incognito of the axilla, drug eruption, pustular psoriasis, bacterial infection, subcorneal pustular dermatosis, pemphigus foliaceous, dermatitis herpetiform, and linear IgA bullous dermatosis. Clinical correlation, histological evaluation, and demonstrating IgA autoantibodies in the epidermis are enough to differentiate IgA pemphigus from another possible differential diagnosis. Classic subcorneal pustular dermatosis is indistinguishable from the subcorneal pustular type of IgA pemphigus clinically and histologically, again DIF is essential in establishing the diagnosis of IgA pemphigus by showing the IgA deposits on adhesion molecules of the keratinocytes.^13^

Although the course of IgA pemphigus is milder than IgG‐driven pemphigus, it is common to have recurrent eruptions at the sites of previous lesions, for which prolonged treatment with dapsone and steroids may be required to prevent such recurrences.^1^ Dapsone has been considered the drug of choice for IgA pemphigus, alternatively, drugs like sulphapyridine, colchicine, acitretin, and photochemotherapy may be used.^2^ A low to medium dose of prednisolone may be added to the aforementioned medications for control of widespread acute eruptions. In a minority of cases, patients are recalcitrant to dapsone therapy and do not respond to the alternative medications as in our case, such recalcitrant cases have been treated successfully with phototherapy, apremilast, and alitretinoin.^14^, ^15^, ^16^ Also, several case reports highlighted the successful treatment of refractory IgA pemphigus with adalimumab injection alone or in combination with other systemic medication, detailed data provided in Table 1.^6^, ^17^ The previous studies didn't define the exact time of tapering or discontinuing adalimumab. In our case, trying to taper the injection slowly was accompanied by new eruptions at the sites of the previous lesions. The author recommends continuing Adalimumab injection as in the case of dapsone treatment until complete remission continuing adalimumab injection as in the case of dapsone treatment until complete remission for a long time and then trying the chance of reducing the dose of the drug, with caution.

**TABLE 1:** Detailed recalcitrant cases of IgA pemphigus treated with adalimumab.

Characteristic	Case 1	Case 2	Case 3 (our case)
Age	41‐year‐old	57‐year‐old	50‐year‐old
Sex	Male	Male	Male
Indication for adalimumab	Not responded to multiple drugs including dapsone, acitretin, UVB, topical and systemic corticosteroid, methotrexate	Not responded to multiple drugs including prednisone, methotrexate, isotretinoin, acitretin, mycophenolate mofetil, and rituximab. Partial response to dapsone which was discontinued due to complications	Not responded to multiple drugs including dapsone, systemic corticosteroid, and topical corticosteroid
Adjuvant drug with Adalimumab	Mycophenolate mofetil 1 g daily	Adalimumab alone	Adalimumab alone
Onset of effect	The effect was observed after 3 weeks	The effect was observed after 3 weeks with nearly complete clearance after 6 months	The effect was seen after 4 weeks and remarkable clearance of lesions was observed after 4 months
Duration of treatment	Not provided	Not provided	More than 2 years and continued until writing this paper
Side effects during treatment	No side effects were observed	No side effects were observed	No side effects were observed
Guidelines to taper or cease the medication	Not provided	Not provided	Because of the rarity of cases of recalcitrant IgA pemphigus treated with adalimumab and no clear guidelines to taper or discontinue the drug in the previously reported cases, The author recommends continuing adalimumab until complete remission for a long time and then trying the chance of reducing the dose of the drug, with caution
Reference Number	^17^	^6^	N/A

Adalimumab injection of 40 mg every 2 weeks was successful in clearing the lesions in our case after 4 months. The mechanism of action of adalimumab in treating IgA pemphigus is thought to be due to the effect of TNF‐alpha inhibitor on preventing neutrophilic infiltration of the skin.^18^ Long‐term follow‐up is needed to know the time of tapering, the time of discontinuing the medication, and the recurrence rate. IgA pemphigus heals without scarring, complications may occur due to prolonged treatment with corticosteroids, dapsone, or other alternative medications.

## 5 CONCLUSION

IgA pemphigus is a rare type of pemphigus group, that presents mainly as pruritic, annular, crusted plaques of the axilla or the inguinal region. The drug of choice in treating IgA pemphigus is dapsone but in some cases may be resistant and not respond to dapsone or other conventional alternative drugs. Here we report a successful treatment of a 50‐year‐old male patient with adalimumab injection who was showing poor response to regular dapsone intake throughout 2 years. Long‐term follow‐up is needed to know the time of tapering, the time of discontinuing the medication, and the recurrence rate.

## AUTHOR CONTRIBUTIONS

**Saman Al‐Zahawi:** Writing – original draft; writing – review and editing. **Kambiz Kamyab:** Supervision; visualization. **Kamran Balighi:** Conceptualization; data curation; supervision; writing – review and editing.

## FUNDING INFORMATION

None.

## CONFLICT OF INTEREST STATEMENT

None.

## CONSENT

Written informed consent was obtained from the patient to publish this report in accordance with the journal's patient consent policy.

## Data Availability

Data available will be provided on request due to privacy/ethical restrictions.
